# Comparison of *Clostridioides difficile* Infection Incidence in Cirrhotic Patients Receiving Secondary Spontaneous Bacterial Peritonitis Prophylaxis With Ciprofloxacin vs. Trimethoprim-Sulfamethoxazole

**DOI:** 10.14740/gr2155

**Published:** 2026-08-26

**Authors:** Allen Barbarovich, Rahul Patel, Hardeep Singh, Jake Slaton, Nelson A. Royall

**Affiliations:** a Northeast Georgia Medical Center, Gainesville, GA 30501, USA; b GME Research, Northeast Georgia Medical Center, Gainesville, GA 30501, USA; c Department of Surgery, Northeast Georgia Medical Center, Gainesville, GA 30501, USA

**Keywords:** Spontaneous bacterial peritonitis, Cirrhosis, Secondary prophylaxis, *Clostridioides difficile* infection, Ciprofloxacin, Trimethoprim-sulfamethoxazole, Retrospective cohort study

## Abstract

**Background:**

Spontaneous bacterial peritonitis (SBP) remains a life-threatening complication in patients with cirrhosis and ascites. Preventing recurrence is a major clinical objective, and prophylactic antibiotic strategies are routinely implemented. Ciprofloxacin and trimethoprim-sulfamethoxazole (TMP-SMX) are two widely used alternatives to norfloxacin, especially in regions where norfloxacin is not available. However, comparative data on adverse outcomes such as *Clostridioides difficile* infection (CDI) and mortality remain scarce. This retrospective cohort study examined the incidence of CDI and mortality in cirrhotic patients receiving ciprofloxacin or TMP-SMX for secondary SBP prophylaxis.

**Methods:**

A total of 6,948 patient encounters were analyzed. Ciprofloxacin was used in 75.4% (5,236) and TMP-SMX in 24.6% (1,712) of cases.

**Results:**

CDI occurred in 0.4% (21) of ciprofloxacin recipients and 0.6% (11) of TMP-SMX recipients. Mortality rates were 0.3% and 0.6%, respectively. A generalized estimating equation (GEE) model showed no statistically significant difference in CDI risk (odds ratio (OR) = 0.623; 95% confidence interval (CI): 0.299–1.296; P = 0.206) or mortality (OR = 0.539; 95% CI: 0.158–1.844; P = 0.320). These findings suggest no statistically significant difference in adverse outcomes between the two regimens, although low event rates may have limited the statistical power of the analysis.

**Conclusion:**

No statistically significant differences in CDI incidence or all-cause mortality were observed between prophylactic regimens. However, low event rates resulted in wide confidence intervals, limiting precision and precluding conclusions regarding comparative effectiveness or equivalence. Larger studies are warranted to validate these observations.

## Introduction

Spontaneous bacterial peritonitis (SBP) remains one of the most clinically significant infectious complications of decompensated cirrhosis, particularly among patients with ascites, and is associated with substantial morbidity, frequent recurrence, and significant mortality despite advances in care [1]. SBP develops mechanistically via bacterial translocation from the intestinal lumen into mesenteric lymph nodes and ascitic fluid, a process facilitated by cirrhosis-related immune dysfunction, increased intestinal permeability, and alterations in gut microbiota composition [2]. Once an initial episode occurs, recurrence rates are strikingly high—reported between 40% and 70% within 1 year—prompting strong recommendations for long-term secondary antibiotic prophylaxis in major hepatology guidelines. Mortality rates for recurrent episodes approach 50%–70% without antibiotic prophylaxis [2, 3].

Historically, norfloxacin has served as the cornerstone of secondary SBP prophylaxis, supported by early randomized controlled trials demonstrating substantial reductions in recurrence rates and improved survival [4, 5]. However, norfloxacin is not available in the United States, and concerns regarding antimicrobial resistance and ecological consequences of long-term fluoroquinolone exposure have led clinicians to adopt alternative regimens [6, 7]. Ciprofloxacin, a second-generation fluoroquinolone with enhanced systemic bioavailability and broader tissue penetration, has emerged as a commonly used substitute, supported by both randomized and observational studies demonstrating efficacy in preventing SBP recurrence [8, 9]. Similarly, trimethoprim-sulfamethoxazole (TMP-SMX), a cost-effective oral antibiotic with broad-spectrum antimicrobial activity, has been shown to provide comparable prophylactic benefit in several comparative studies and is frequently utilized in settings of fluoroquinolone resistance or intolerance [10].

A growing body of literature has evaluated the comparative effectiveness of antibiotic regimens for SBP prophylaxis. Network meta-analyses and systematic reviews suggest that fluoroquinolones and TMP-SMX offer similar efficacy in reducing SBP recurrence and may confer comparable survival benefits [1, 6, 11]. However, these studies primarily focus on the prevention of SBP and overall mortality, with relatively limited attention to adverse infectious consequences of long-term antibiotic exposure. This gap is clinically relevant, as cirrhotic patients represent a uniquely vulnerable population with immune dysregulation, frequent healthcare exposure, and repeated antibiotic use, all of which increase susceptibility to secondary infections [2, 12].

Among these secondary complications, *Clostridioides difficile* infection (CDI) has emerged as a major international healthcare concern. CDI is a leading cause of healthcare-associated diarrhea and is associated with significant morbidity, mortality, and healthcare costs [13]. CDI pathogenesis is closely linked to disruption of the normal gut microbiome, typically following exposure to antibiotics that alter microbial diversity and facilitate colonization by toxigenic *C. difficile* strains [2]. Certain antibiotic classes—particularly fluoroquinolones, clindamycin, and broad-spectrum cephalosporins—have been consistently associated with higher CDI risk in both inpatient and community settings [14].

Fluoroquinolones are of particular concern due to their profound impact on intestinal microbiota and their association with hypervirulent *C. difficile* strains, such as the NAP1/BI/027 lineage [2]. Epidemiologic studies and meta-analyses have demonstrated a strong association between fluoroquinolone exposure and CDI, with ORs significantly higher than many other antibiotic classes [14]. In cirrhotic populations, this risk may be further amplified due to baseline dysbiosis, impaired immune function, and frequent hospitalizations [2]. Moreover, cirrhosis itself has been identified as an independent risk factor for CDI and is associated with worse outcomes, including increased mortality, longer hospital stays, and higher rates of recurrence [8].

In contrast, TMP-SMX has a less clearly defined association with CDI. While it also alters gut flora, its impact appears to be less pronounced than that of fluoroquinolones, and epidemiologic data linking TMP-SMX to CDI are inconsistent [2]. Some observational studies suggest a modest increase in risk, whereas others have not demonstrated a significant association [2]. This relative uncertainty, combined with its comparable efficacy in SBP prophylaxis, has led to increasing interest in TMP-SMX as a potentially safer and more economically prudent alternative in terms of CDI risk.

Despite the widespread use of ciprofloxacin and TMP-SMX for secondary SBP prophylaxis, there remains a paucity of data directly comparing their safety profiles, particularly with respect to CDI incidence. Existing studies have largely focused on SBP recurrence and survival outcomes, with limited attention to antibiotic-associated complications [1, 6, 11]. Additionally, recent data suggest that long-term antibiotic prophylaxis in cirrhosis may alter the spectrum of infections, increasing the incidence of non-SBP infections and promoting antimicrobial resistance [12, 15]. These findings highlight the need for a more nuanced evaluation of prophylactic strategies that balance efficacy with downstream infectious risks.

The clinical relevance of CDI in cirrhotic patients cannot be overstated. CDI in this population is associated with increased rates of hepatic decompensation, acute kidney injury, and mortality [8]. Furthermore, management of CDI in cirrhosis is often complicated by altered pharmacokinetics, co-existing organ dysfunction, and competing infectious risks [13]. Given these challenges, minimizing CDI risk through judicious antibiotic selection represents an important component of comprehensive cirrhosis care.

In addition to CDI risk, antibiotic selection may also influence broader clinical outcomes, including all-cause mortality. While mortality in cirrhotic patients is multifactorial and driven by the severity of underlying liver disease, infections remain a major contributor to decompensation and death [2]. The choice of prophylactic antibiotic may therefore have indirect effects on survival through its impact on infection patterns, antimicrobial resistance, and microbiome integrity [1, 12].

Although prior studies have demonstrated similar efficacy of ciprofloxacin and TMP-SMX in preventing SBP recurrence, direct comparisons of their impact on CDI incidence and overall mortality are lacking [10]. This represents a critical knowledge gap, particularly in the United States where norfloxacin is not routinely available and clinicians must choose between alternative agents. Given the increasing recognition of CDI as a significant complication in cirrhosis and the differential risk profiles of antibiotic classes, comparative data are needed to inform evidence-based decision-making.

The purpose of this study is to retrospectively evaluate the incidence of CDI and all-cause mortality among cirrhotic patients receiving secondary SBP prophylaxis with ciprofloxacin or TMP-SMX. We hypothesized that there is a significantly increased risk for CDI in cirrhotic patients utilizing ciprofloxacin compared to TMP-SMX for SBP prophylaxis. We also hypothesized that patients utilizing ciprofloxacin would have a significantly elevated mortality compared to those using TMP-SMX.

## Methods

This study employed a retrospective cohort using existing electronic health record (EHR) data from the COSMOS database, a de-identified database created in collaboration with a community of Epic health systems representing more than 300 million patient records from over 1,762 hospitals and 40,700 clinics from January 1, 2018 to May 31, 2024. Adult patients (≥ 18 years) with a documented diagnosis of cirrhosis ascites (ICD-10 K70.31) and a history of SBP (ICD-10 K65.2) who received ciprofloxacin (500 mg daily) or TMP-SMX (800/160 mg daily) for secondary prophylaxis were included. Patients were identified through ICD-10 diagnostic codes associated with cirrhosis and SBP. Medication exposure was determined using prescriptions. Patients were excluded if they had evidence of concurrent prophylactic use of both agents (medication use less than 30 days), prior diagnosis of CDI (ICD-10 A04.72, A04.71) within 6 months of the index prescription, active malignancy requiring chemotherapy, or incomplete demographic or medication data.

The index date was defined as the date of initiation of ciprofloxacin or TMP-SMX for secondary SBP prophylaxis. Follow-up began on the index date and continued until discontinuation of prophylaxis, occurrence of the outcome of interest, death, loss to follow-up, or the end of the study period whichever occurred first.

CDI was defined as laboratory-confirmed *Clostridioides difficile* infection occurring during follow-up. Mortality was defined as all-cause in-hospital mortality occurring during the observation period.

Outcomes were analyzed at the encounter level because patients could contribute multiple eligible prophylaxis episodes. Generalized estimating equations (GEEs) clustered by patient identifier accounted for correlation among repeated observations.

A total of 6,948 eligible encounters contributed by 4,295 unique patients were included in the analysis. Patients contributed a median of one encounter (interquartile range (IQR) 1–2), and 32.9% contributed more than one encounter. Because observations were not independent, analyses were conducted at the encounter level using GEEs clustered by unique patient identifier.

Demographic variables (age, sex, race, and ethnicity), clinical characteristics, and medication duration were extracted. The primary outcome was the occurrence of CDI after initiating SBP prophylaxis. CDI was confirmed through laboratory identification of *C. difficile* toxin or polymerase chain reaction (PCR). The secondary outcome was all-cause in-hospital mortality during the prophylaxis period.

A GEE with a logit link function was employed to model binary outcomes (CDI and mortality) under the assumption that the outcomes follow a binomial distribution. The GEE accounted for repeated patient encounters by clustering on a unique patient identifier and used an exchangeable correlation structure to assume equal correlation across repeated observations. ORs with 95% CIs and P-values were reported. Statistical significance was defined as P < 0.05. All analyses were conducted using R6 (version 4.4.1) in the open-source RStudio IDE17 (Posit Software, PBC, Boston, MA, USA) with the following packages: *tidyverse18*, *gt19*, *gtsummary20*, *janitor21,* and *geepack22*. The study was deemed exempt by the Brenau University Institutional Review Board (2200752-1). This study was conducted in compliance with the ethical standards of the responsible institution on human subjects as well as with the Helsinki Declaration.

## Results

A total of 6,948 eligible encounters from 4,295 unique patients met the inclusion criteria and were included in the analysis. Patients contributed a median of one encounter (IQR 1–2), with 32.9% contributing more than one encounter. Among all encounters, 5,236 (75.4%) received ciprofloxacin and 1,712 (24.6%) received TMP-SMX for secondary SBP prophylaxis. The median age was 54 years (IQR 45–61), and 30.1% of patients were female. Racial distribution was predominantly White (78.6%), followed by Black or African American (9.4%), American Indian/Alaska Native (3.9%), Asian (1.6%), etc. Hispanic or Latino(a) ethnicity was reported in 19.1% of encounters (Table 1). The overall incidence of CDI was 0.5% (32 cases), and all-cause mortality occurred in 0.3% (20 cases).

**Table 1:** Baseline Characteristics of the Study Population

Characteristic	Value
Total encounters	6,948
Age, median (IQR)	54 (45, 61)
Female	30.1%
White	78.6%
Black or African American	9.4%
American Indian/Alaska Native	3.9%
Asian	1.6%
Other	6.1%
Multiracial	14.1%
Not Hispanic or Latino	80.9%
Hispanic or Latino	19.1%
CDI cases	32 (0.5%)
Mortality	20 (0.3%)
Ciprofloxacin	5,236 (75.4%)
TMP-SMX	1,712 (24.6%)
Medication duration (days)	77 (36, 165)

IQR: interquartile range; CDI: *Clostridioides difficile* infection; TMP-SMX: trimethoprim- sulfamethoxazole.

When stratified by the medication group, the ciprofloxacin cohort had a CDI rate of 0.4% (21 cases), while the TMP-SMX cohort had a slightly higher rate at 0.6% (11 cases). Mortality was reported in 17 ciprofloxacin patients (0.3%) and fewer than 11 TMP-SMX patients. Median prophylaxis duration was similar: 77 days (IQR 36–167) for ciprofloxacin and 73 days (IQR 37–160) for TMP-SMX (Table 2).

**Table 2:** Group Comparison by Medication

Characteristic	Ciprofloxacin (n = 5,236)	TMP-SMX (n = 1,712)
Age, median (IQR)	54 (45, 61)	53 (44, 60)
Female	29.7%	31.3%
White	79.3%	76.4%
Black or African American	9.2%	9.7%
American Indian/Alaska Native	3.3%	5.8%
Asian	1.5%	1.7%
Other	6.2%	5.8%
Multiracial	14.1%	14.3%
Not Hispanic or Latino	80.3%	82.6%
Hispanic or Latino	19.7%	17.4%
CDI cases	21 (0.4%)	11 (0.6%)
Mortality	17 (0.3%)	< 11
Medication duration (days)	77 (36, 167)	73 (37, 160)

IQR: interquartile range; CDI: *Clostridioides difficile* infection; TMP-SMX: trimethoprim- sulfamethoxazole.

The GEE model showed no statistically significant difference in the risk of CDI between the two groups. The OR for CDI in patients receiving ciprofloxacin compared to TMP-SMX was 0.623 (95% CI: 0.299–1.296; P = 0.206). Similarly, there was no statistically significant difference in mortality (OR = 0.539; 95% CI: 0.158–1.844; P = 0.320). These results are summarized in Tables 3 and 4.

**Table 3:** GEE Model: CDI Risk

Comparison	OR	95% CI	P-value
Ciprofloxacin vs. TMP-SMX	0.623	(0.299, 1.296)	0.206

GEE: generalized estimating equation; CDI: *Clostridioides difficile* infection; OR: odds ratio; CI: confidence interval; TMP-SMX: trimethoprim-sulfamethoxazole.

**Table 4:** GEE Model: Mortality Risk

Comparison	OR	95% CI	P-value
Ciprofloxacin vs. TMP-SMX	0.539	(0.158, 1.844)	0.320

GEE: generalized estimating equation; OR: odds ratio; CI: confidence interval; TMP-SMX: trimethoprim-sulfamethoxazole.

Although point estimates suggested a slightly lower risk of adverse outcomes in the ciprofloxacin group, the CIs were wide, and the findings were not statistically significant, likely due to the low event rates for both outcomes.

## Discussion

SBP is a life-threatening complication of cirrhosis characterized by high recurrence and mortality rates, necessitating long-term secondary prophylaxis after an index episode or in select patients with depressed ascites albumin (< 1.5 g/dL) [16]. In this retrospective cohort study of 6,948 cirrhotic patient encounters, we found no statistically significant difference in the incidence of CDI or all-cause mortality between patients receiving ciprofloxacin and those receiving TMP-SMX for secondary SBP prophylaxis. Although point estimates suggested a lower CDI rate with ciprofloxacin (0.4% vs. 0.6%), this did not reach statistical significance (OR = 0.623; 95% CI: 0.299–1.296; P = 0.206). Mortality similarly did not differ significantly (OR = 0.539; 95% CI: 0.158–1.844; P = 0.320).

Important sources of residual confounding could not be fully assessed because detailed clinical variables were unavailable or incompletely captured within the de-identified database. These include liver disease severity measures such as model for end-stage liver disease (MELD) or Child-Pugh scores, renal dysfunction, proton pump inhibitor exposure, prior antibiotic use, hospitalization frequency, use of rifaximin or lactulose, and other comorbid conditions associated with CDI risk. Consequently, the observed associations should be interpreted as hypothesis-generating rather than causal.

The comparison between ciprofloxacin, a fluoroquinolone, and TMP-SMX is clinically relevant due to evolving prescribing patterns and unavailability of norfloxacin in the United States [17]. Fluoroquinolones have long been a mainstay for SBP prophylaxis due to their activity against Gram-negative enteric organisms and favorable pharmacokinetics [4]. However, concerns regarding antimicrobial resistance and CDI risk have prompted increased use of TMP-SMX as an alternative [18]. Fluoroquinolones are known to disrupt intestinal microbiota and have been associated with increased CDI risk in multiple populations [19, 20], whereas TMP-SMX may exert a comparatively less pronounced microbiome dysbiosis [21]. Therefore, understanding whether these theoretical risks translate into clinically meaningful differences in cirrhotic populations is essential.

Prior randomized controlled trials and observational studies have demonstrated comparable efficacy between fluoroquinolones and TMP-SMX for SBP prophylaxis [22, 23]. Studies comparing TMP-SMX with norfloxacin have shown similar rates of SBP recurrence and survival [22], while weekly ciprofloxacin has been shown to be non-inferior to daily norfloxacin in preventing SBP [24]. Meta-analyses similarly suggest no clear superiority of one regimen over another for SBP prevention [25]. With respect to CDI, fluoroquinolones have consistently been identified as high-risk antibiotics due to their association with hypervirulent strains and microbiome disruption [20, 26]. Reported CDI incidence in cirrhotic populations receiving prophylaxis varies, typically ranging from 1% to 5% depending on study design and patient characteristics [27, 28]. Notably, the CDI incidence observed in our cohort (0.5%) is lower than previously reported ranges. This discrepancy may reflect differences in antimicrobial stewardship practices, diagnostic criteria, or patient selection. Importantly, despite established associations between fluoroquinolones and CDI, our findings did not demonstrate a statistically significant increase in CDI risk with ciprofloxacin relative to TMP-SMX.

The observed trend toward lower CDI and mortality with ciprofloxacin, although not statistically significant, may still represent a clinically relevant difference. The wide confidence intervals observed in our analysis reflect limited precision due to low event rates (32 CDI events and 20 deaths), increasing the likelihood of type II error. From a clinical standpoint, even small differences in CDI incidence may be meaningful in cirrhotic patients, who are particularly vulnerable to infectious complications and poor outcomes [29]. Therefore, clinicians should interpret these findings cautiously, recognizing that underpowered studies may fail to detect important differences.

### Strengths and limitations

Strengths of this study include its large sample size, multicenter design, and use of GEE modeling to account for repeated encounters. The use of real-world data enhances generalizability.

Limitations include the retrospective design, potential residual confounding, and reliance on administrative coding and laboratory data. Two major challenges include identifying whether the antibiotic was prescribed specifically for prophylaxis, which was targeted by criteria specifying the minimum duration of antibiotic use for 30 days, as well as compliance that is difficult to quantify with the available data. Additionally, low event rates limited statistical power and precision. Data on the duration of prophylaxis, adherence, and microbiologic resistance patterns were not fully captured. This study focused specifically on adverse outcomes associated with secondary SBP prophylaxis, including CDI and mortality, and did not evaluate SBP recurrence. Consequently, these findings should not be interpreted as evidence supporting the comparative efficacy of ciprofloxacin versus TMP-SMX for the prevention of recurrent SBP.

A prospective randomized trial comparing these therapies is likely to be cost-prohibitive; therefore, it is re-assuring that with both regimens the CDI incidence is rare relative to population incidence. Large, pooled analyses or registries will be useful to increase event rates and improve estimate precision. Microbiome studies evaluating differential effects of these agents on gut flora in cirrhotic patients should also guide future research into optimizing the clinical management of this patient cohort. Risk-stratified analyses in patients with prior CDI or advanced liver disease and integration of resistance data to guide optimal prophylactic strategies in evolving antimicrobial landscapes should be considered.

### Conclusions

In this large retrospective cohort, ciprofloxacin and TMP-SMX demonstrated similar rates of CDI and all-cause mortality when used for secondary SBP prophylaxis. While no significant differences were identified, low event rates and potential confounding variables limit definitive conclusions. These findings should be considered hypothesis-generating and require confirmation in larger studies with greater statistical power.

## Data Availability

The data supporting the findings of this study are available from the corresponding author upon reasonable request.

## Abbreviations

CDI: *Clostridioides difficile* infection
CI: confidence interval
EHR: electronic health record
GEE: generalized estimating equation
IQR: interquartile range
MELD: model for end-stage liver disease
OR: odds ratio
PCR: polymerase chain reaction
SBP: spontaneous bacterial peritonitis
TMP-SMX: trimethoprim-sulfamethoxazole
